# Formation and Dynamics of Waves in a Cortical Model of Cholinergic Modulation

**DOI:** 10.1371/journal.pcbi.1004449

**Published:** 2015-08-21

**Authors:** James P. Roach, Eshel Ben-Jacob, Leonard M. Sander, Michal R. Zochowski

**Affiliations:** 1 Neuroscience Graduate Program, University of Michigan, Ann Arbor, Michigan, United States of America; 2 School of Physics and Astronomy, Tel-Aviv University, Tel Aviv, Israel; 3 Center for Theoretical Biological Physics, and Department of Biochemistry and Cell Biology, Rice University, Houston, Texas, United States of America; 4 Department of Physics & Center for Studies of Complex Systems, University of Michigan, Ann Arbor, Michigan, United States of America; 5 Biophysics Program, University of Michigan, Ann Arbor, Michigan, United States of America; Indiana University, UNITED STATES

## Abstract

Acetylcholine (ACh) is a regulator of neural excitability and one of the neurochemical substrates of sleep. Amongst the cellular effects induced by cholinergic modulation are a reduction in spike-frequency adaptation (SFA) and a shift in the phase response curve (PRC). We demonstrate in a biophysical model how changes in neural excitability and network structure interact to create three distinct functional regimes: localized asynchronous, traveling asynchronous, and traveling synchronous. Our results qualitatively match those observed experimentally. Cortical activity during slow wave sleep (SWS) differs from that during REM sleep or waking states. During SWS there are traveling patterns of activity in the cortex; in other states stationary patterns occur. Our model is a network composed of Hodgkin-Huxley type neurons with a M-current regulated by ACh. Regulation of ACh level can account for dynamical changes between functional regimes. Reduction of the magnitude of this current recreates the reduction in SFA the shift from a type 2 to a type 1 PRC observed in the presence of ACh. When SFA is minimal (in waking or REM sleep state, high ACh) patterns of activity are localized and easily pinned by network inhomogeneities. When SFA is present (decreasing ACh), traveling waves of activity naturally arise. A further decrease in ACh leads to a high degree of synchrony within traveling waves. We also show that the level of ACh determines how sensitive network activity is to synaptic heterogeneity. These regimes may have a profound functional significance as stationary patterns may play a role in the proper encoding of external input as memory and traveling waves could lead to synaptic regularization, giving unique insights into the role and significance of ACh in determining patterns of cortical activity and functional differences arising from the patterns.

## Author Summary

Within the brain, networks of neurons with relatively stable anatomical connections will rapidly shift patterns of neural activation when exposed to changing chemical environments. A classic example of this is the transition from high frequency to low frequency electrical patterns as animals move from waking to slow wave sleep. The neurotransmitters responsible for these transitions act through changes in neural excitability and their effect on network patterns arises through an interaction of stable network features and dynamic neural properties. In this paper we study how network features, namely excitatory/ inhibitory balance, and dynamic neural properties, the presence or absence of spike-frequency adaptation, shape large-scale patterns in network activity. We focus on the neuromodulator largely responsible for the transition between waking and slow-wave sleep, acetylcholine. We show that inhibition and SFA work together to set the speed of activity propagation through a network by altering the spatial (inhibition) and temporal (SFA) extent of neural activity. By scanning these parameters a variety of dynamical regimes can be created.

## Introduction

The difference between cortical activity patterns during waking, rapid eye movement sleep (REM), and slow wave sleep (SWS) is striking. During waking and REM sleep low amplitude, high frequency EEG and local field potential (LFP) recordings suggest that cortical population dynamics are localized. Conversely, in SWS, the dynamics enter a slow (∼ 1 Hz) oscillation state where individual neurons oscillate between a high frequency (up) state and periods of quiescence (down state) [1, 2]. The functional role of high frequency local activation (i.e. waking or REM state) has been linked to attention and working memory [3–5], while traits of SWS have been related to synaptic homeostasis and sleep pressure [2, 6–8]. Both of these dynamic patterns can be thought of as upstates, but with differing lengths.

**Fig 1:**
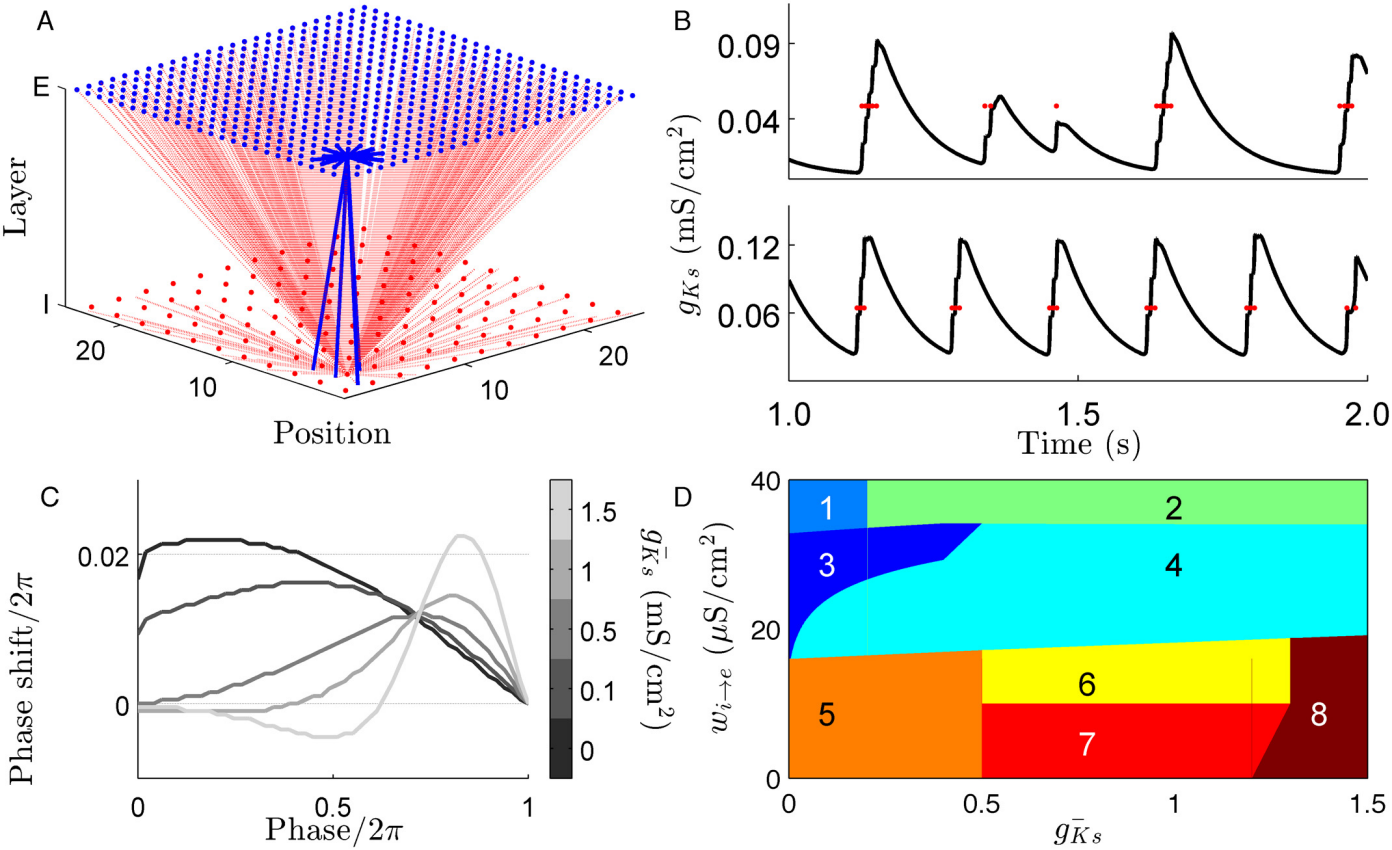
Cortical network model of cholinergic modulation. (*A*.) Our network model consists of a square lattice split into an excitatory and an inhibitory layer. A connectivity scheme balancing short-range excitation and global inhibition was used to mimic the lateral inhibition motif seen in many areas of the cerebral cortex. (*B*.) Examples of spike-frequency adaptation (SFA) induced by the slow potassium conductance are shown for $\bar{g_{Ks}}$ = 0.75 mS/cm^2^ (top) and $\bar{g_{Ks}}$ = 1.5 mS/cm^2^ (bottom). The red dots indicate the spike times of the neuron in question and illustrate the the offset of an upstate corresponds to the maximal level of $\bar{g_{Ks}}$. (*C*.) The phase response curve of individual neurons shifts from type 1 to type 2 as $\bar{g_{Ks}}$ increases. (*D*.) An illustration of the dynamics sampled by scanning inhibitory strength, (*w*_*i* → *e*_), and $\bar{g_{Ks}}$. In a general sense, the spatial scope of activity is determined by the excitatory/ inhibitory balance and the temporal scope of activity is determined by the strength of SFA. Key: (1) quiescent (2) mixed dynamics, (3) stationary bump (4) traveling bump (5) global high frequency activity (6) multiple interacting bumps (7) planar wave (8) global burst.

Acetylcholine (ACh) is a neurotransmitter that governs the cortical dynamics associated with arousal and sleep state. Levels of ACh rise during the transition from NREM sleep to waking or REM sleep. ACh acts through two pathways, the nicotinic receptor and the muscarinic receptor. The nicotinic receptor directly depolarizes cells while the muscarinic suppresses voltage-gated potassium channels. Inactivation of these channels, and the current associated with them (the M-current), changes the intrinsic excitability of neurons. Experiments have shown that ACh modulates neural excitability in two ways: (1) ACh reduces spike frequency adaptation (SFA) mediated by the M-current and increases the slope of the neural spike frequency-current (f-I) curve [9, 10], and (2) it induces changes in the synchronization properties of neurons via the phase response curve (PRC) [11, 12]. ACh induces a shift from a biphasic type 2 PRC to monophasic type 1 PRC (Fig 1C). It has been previously shown that networks of type 1 neurons are asynchronous while those of type 2 neurons are highly synchronous [12].

The aim of this paper is to elucidate how cholinergic modulation interacts with network connectivity structure to form various patterns of network activation obtained experimentally. To do so we use simulations of a conductance-based (Hodgkin-Huxley) cortical network model including cholinergic modulation [13] and a mexican hat type of connectivity scheme that was experimentally observed in various cortical areas [14, 15].

We demonstrate how regulation of SFA in conjunction with the balance between excitation and inhibition leads to various network dynamics. We show that ACh driven reduction of SFA in model networks with lateral inhibition is responsible for the transition from moving to stationary dynamics and E/I balance is responsible for switching between highly local and global dynamics. We then study the properties of the two states and their transition. Functionally, the high ACh state is far more sensitive to heterogeneities in network structure than the traveling wave state. In this neuron model, SFA and PRC effects occur over different ranges of ACh, which leads to three distinct functional regimes. Further, we show that both SFA and E/I balance shape network activity by setting the spatial (E/I balance) and temporal (SFA) extent of network upstates.

## Models

### Neuron model

We use a conductance-based model of cholinergic modulation in pyramidal cells using Hodgkin-Huxley type gating dynamics for active conductances [13]. The membrane voltage dynamics are described by:

$$\begin{array}{c} c_{m}\frac{dV}{dt}=-{m_{\infty }}^{3}h\bar{g_{Na}}\left(V-E_{Na}\right)-n^{4}\left(V\right)\bar{g_{K_{dir}}}\left(V-E_{K}\right)\\ -s\bar{g_{Ks}}\left(V-E_{K}\right)-\bar{g_{l}}\left(V-E_{l}\right)+I^{tune}-I^{syn} \end{array}$$ (1)

The gating variables *m*, *h*, *n*, and *s* represent the sodium conductance, the effective blockage of sodium current and potassium conductances respectively. In the case of *h*, *n*, and *s* dynamics of the form:

$$dx/dt=\frac{\left(x-x_{\infty }\left(V\right)\right)}{\tau_{x}\left(V\right)}.$$

The function *x*_∞_(*V*) = 1/(1 + exp((*α*_*x*_ − *V*)/*β*_*x*_) represents the steady state gating for each conductance and gating time constant, *τ*_*x*_, is constant for *s*, *τ*_*s*_ = 75, is governed by *τ*_*x*_ = 0.37 + *D*_*x*_/(1 + exp((*γ*_*x*_ + *V*)/*ɛ*_*x*_)) for *h* and *n*. The *s* variable corresponds to the slow potassium current which is ultimately responsible for the shift in neural excitability mediated by ACh. Adjusting the magnitude of this current (i.e. varying the parameter $\bar{g_{Ks}}$) changes neuronal excitability characterized by the level of SFA. For low $\bar{g_{Ks}}$ values neurons have a minimal level of SFA. As $\bar{g_{Ks}}$ increases the neurons display high levels of SFA.

The direct input current, *I* ^*tune*^, was adjusted so that all cells fired at 10 Hz in the absence of any synaptic input, independent of $\bar{g_{Ks}}$. The synaptic input to *ith* neuron is given by:

$$I_{i}^{syn}=\sum_{j=1}^{n}A_{i,j}w_{j\to i}K\left(e^{\frac{-(\tilde{t}-\tau_{D})}{\tau_{S}}}-e^{\frac{-(\tilde{t}-\tau_{D})}{\tau_{F}}}\right)\left(V_{i}-E_{syn}\right),$$ (2)

where *A*_*i*,*j*_ is the network adjacency matrix, ${\tilde{t}}_{j}$ is the time of the last spike of neuron *j*, *τ*_*F*_ and *τ*_*S*_ are synaptic time costants and *τ*_*D*_ is the synaptic delay. The parameter *w*_*j* → *i*_ is the synaptic coupling between neurons *i* and *j* based on the respective species of each. *K* is a normalization constant such that the range of each synaptic pulse is ∈ [0, *w*_*j* → *i*_]. Values of the neural parameters were adopted from [12] and are listed in 1. The equations were solved for 5 seconds at 0.05 ms time steps using the 4th order Runge-Kutta algorithm (simulation code is provided in S1 File).

### Network model and measurements of dynamics

We considered networks composed of 625 excitatory and 121 inhibitory neurons evenly distributed over a two-layer lattice of sides *L* = 25 (Fig 1A) with periodic boundaries. The fraction of inhibitory cells, 16%, was close to the 20% seen in the cortex [16] and the dynamics were robust to shifts in inhibitory fraction between 13% to 22% (Supplemental S1 Video to S4 Video). To evenly distribute the inhibitory cells with respect to the excitatory layer the spacing of inhibitory cells, *Grain*_*i*_, was 2.87 lattice units while excitatory cells were spaced *Grain*_*e*_, at 1 unit. We used a center-surround (or lateral inhibition) type network scheme which balances short-range excitation and global inhibition. This is an established model for cortical connectivity [17]. All excitatory neurons were connected to all cells within a radius defined by:

$$R_{xx}=\sqrt{\frac{L^{2}k_{xx}}{\pi N}}$$ (3)

where *k*_*ee*_ = 16 and *k*_*ei*_ = 4. This leads to 20 connections to excitatory and 4 to inhibitory nearest neighbors. Inhibitory neurons were globally connected. Unless otherwise stated the maximum synaptic strengths were 20 μS/cm^2^ for all synapses.

As we will see (Fig 1D) the dynamics which result vary qualitatively depending on the values of the parameters introduced above. It includes cases where spiking is spatially confined (’stationary bump’), where the activity moves (’moving bump’), plane waves of activity, global bursting, etc. To illustrate the character of the dynamics on raster plots cells were sorted by a spatial coordinate given by *S*_*i*_ = *y*_*i*_ + *x*_*i*_/*L* where *x*_*i*_ and *y*_*i*_ are the coordinates of the cell in the lattice.

The speed of moving bumps of activity was calculated by dividing the simulation time into 10 ms bins in which the frequency of all excitatory cells were calculated. For each time bin the center of activity was calculated in a manner similar to a center-of-mass calculation using an algorithm previously described in [18], which accounts for the periodic boundaries of the network. The wave speed was averaged over the final 2.5 s of the simulation run. When averaging wave speed the following cases were excluded because they were not appropriate for our speed measure: where no excitatory cells were active, where more than 300 cells were active within any 10 ms time bin, where the standard deviation of active cells was greater than the mean number of active cells within 10 ms time bins, when more than one bump of activity was stable, or when the network was highly synchronized.

To measure synchronization we used the bursting measure:

$$B=\frac{1}{\sqrt{N}}\left(\frac{\sqrt{\left\langle \tau^{2}\right\rangle -{\left\langle \tau \right\rangle }^{2}}}{\left\langle \tau \right\rangle }-1\right)$$ (4)

where *τ* is the series of inter-spike intervals of all spikes regardless of cell identity and *N* is the total number of spikes [12]. This measure approaches 1 as network activity approaches perfect synchrony. We consider a network to be synchronized when *B* > 0.7 as this value bisects the bimodal distribution of B (S1 Fig).

Heterogeneities were added to the network by multiplying the strength of excitatory to excitatory connections beginning and terminating within a 8 x 8 region of the network by a constant value ranging from 1.005 to 2.5 (i.e., increasing recurrent excitation between 0.5% to 150%). Neurons within the heterogeneity also received an additional 0.5 *μ*A/cm^2^ of direct current during the first 0.5 s of the simulation.

Preference for the heterogeneous region is described by the normalized measure *ϕ* = (*f*_*in*_ − *f*_*out*_)/(*f*_*in*_ + *f*_*out*_) where *f*_*in*_ and *f*_*out*_ are the average frequency of excitatory neurons inside and outside the heterogeneity respectively. *ϕ* ranges between 1, when the only activity is within the heterogeneity, and -1, when all network activity is outside. When measuring network preference for the synaptic heterogeneity *ϕ* was calculated for the last half of the simulation run.

## Results

We used the above model to elucidate how ACh modulation together with the network connectivity properties regulates spatio-temporal dynamics in a system. The level of $\bar{g_{Ks}}$ sets the amount of SFA in each neuron and shortens the length of an upstate both in time and in the number of spikes fired (Fig 1B). Sampling the parameter space defined by $\bar{g_{Ks}}$ and *w*_*i* → *e*_ allows for multiple dynamical regimes to emerge. These range from complete quiescence for excitatory cells at one extreme to globalized network bursts at another (Fig 1D and S1 Fig).

Simulations under a variety of network structures and network sizes yielded qualitatively similar results provided that the radius of inhibitory connections was larger than that of excitatory connections. Reducing *R*_*ie*_ from global to smaller values leads to multiple independent bumps. Note that these dynamics are different from the multiple interacting bumps described in Fig 1D. Implementing a heterogeneous network lattice where neurons are placed at irregular intervals, the degree distribution of neurons is nonuniform, and connections are rewired based on the Watts-Strogatz formalism [19] did not change the the results qualitatively. Removing periodic boundary conditions leads to traveling waves in a circular as opposed to periodic direction (S5 Video).

**Fig 2:**
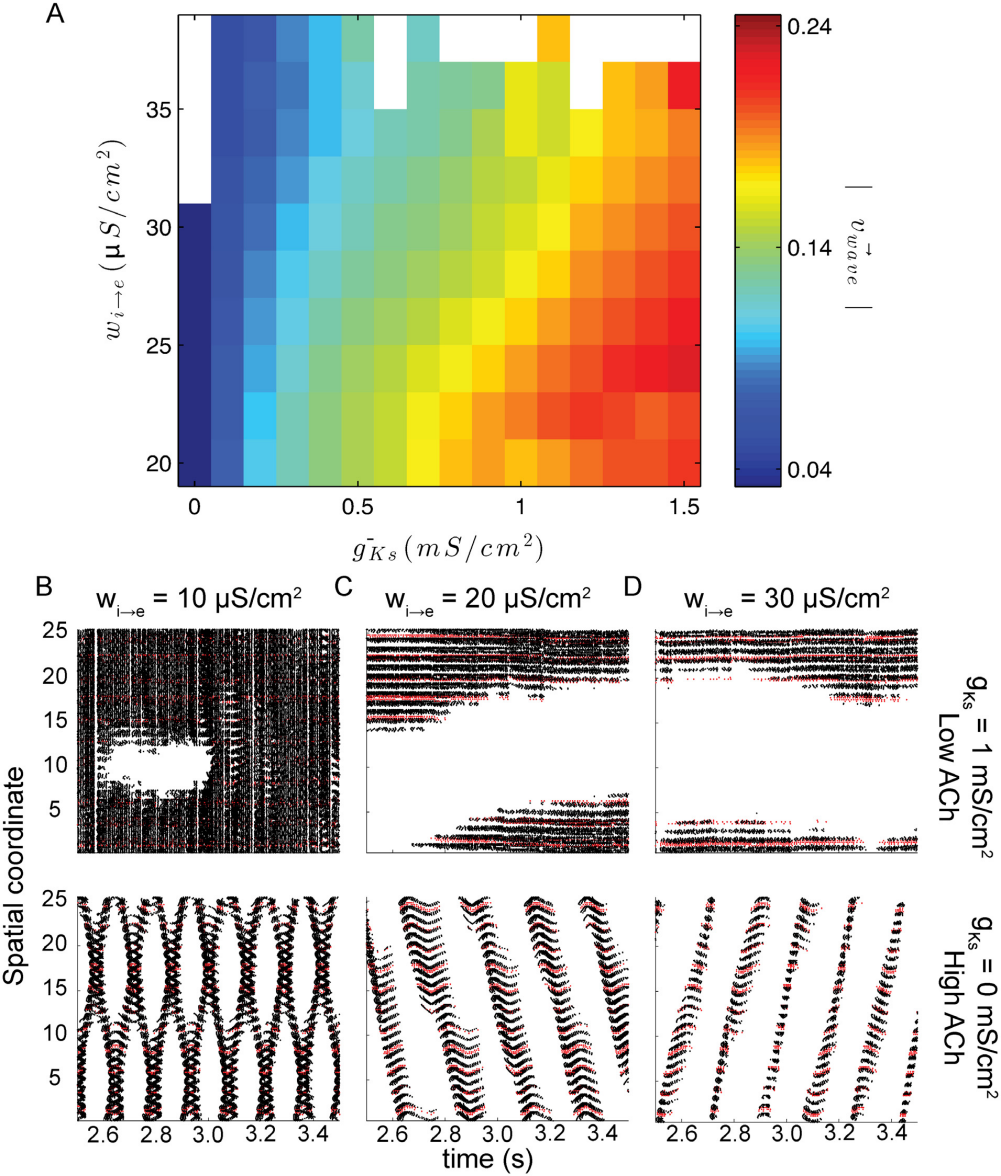
Both E/I balance and SFA level affect the spatio-temporal properties of cortical activities. (*A*.) Within the single bump the levels of $\bar{g_{Ks}}$ and *w*_*i* → *e*_ determine the speed of propagation. However, SFA does have a much larger effect on wave speed than E/I balance. Panels *B*, *C*, and *D* show example raster plots for *w*_*i* → *e*_ = 10, 20, and 30 *μ*S/cm^2^ respectively with $\bar{g_{Ks}}$ = 0 mS/cm^2^on top and $\bar{g_{Ks}}$ = 1 mS/cm^2^ on bottom. In each, black markers represent spikes from excitatory cells and red markers represent those from inhibitory cells. Cells are sorted by spatial coordinate, a measure described in the methods section.

Moderate levels of inhibition (*w*_*i* → *e*_ = 20 *μ*S/cm^2^) generated two distinct classes of dynamics as the level of SFA was changed. When $\bar{g_{Ks}}$ levels are low (which corresponds to a high ACh state) network activity is localized to a restricted area with minimal drift, the stationary bump regime (Fig 2C top). Increasing $\bar{g_{Ks}}$ (or decreasing ACh) leads to a localized traveling wave of activation (a bump) that traverses the entire network space (Fig 2C bottom). Local dynamics are characterized by high frequency, asynchronous spiking. Spike dynamics in the global state are also asynchronous, but oscillate between a high frequency upstate and a low frequency down state. While the level of SFA in the system controls the amount of time that activity remains in any single location, the level of inhibition (E/I balance) defines the size of the portion of the network that is in an upstate at any given time. For low levels of inhibition (*w*_*i* → *e*_ = 10 *μ*S/cm^2^), depending on SFA level, either the whole network is active with quiescent regions emerging periodically (low levels of SFA; $\bar{g_{Ks}}$ = 0 mS/cm^2^; Fig 2B top) or two distinct interacting bumps are stable (high levels of SFA; $\bar{g_{Ks}}$ = 1 mS/cm^2^; Fig 2B bottom). High levels of inhibition (*w*_*i* → *e*_ = 30 *μ*S/cm^2^), however, reduce the spatial extent of activity at any given time (Fig 2D).

The interplay between SFA and inhibitory strength (*w*_*i* → *e*_) is shown in Fig 2A. The SFA level (i.e. the magnitude of $\bar{g_{Ks}}$) is the primary factor in determining the transition between the localized and global activation state. For any given level of inhibition, the transition between stationary and traveling frequency dynamics occurs over a narrow range of $\bar{g_{Ks}}$. The effect of inhibition becomes clearer in the traveling wave regime, where the speed of the wave propagation is slowed by increased inhibitory strength. For strong values of inhibition waves are arrested. The empty squares of Fig 2A indicate parameter values that yield networks where excitatory cells are completely quiescent or involved in network-wide synchronous bursting.

**Fig 3:**
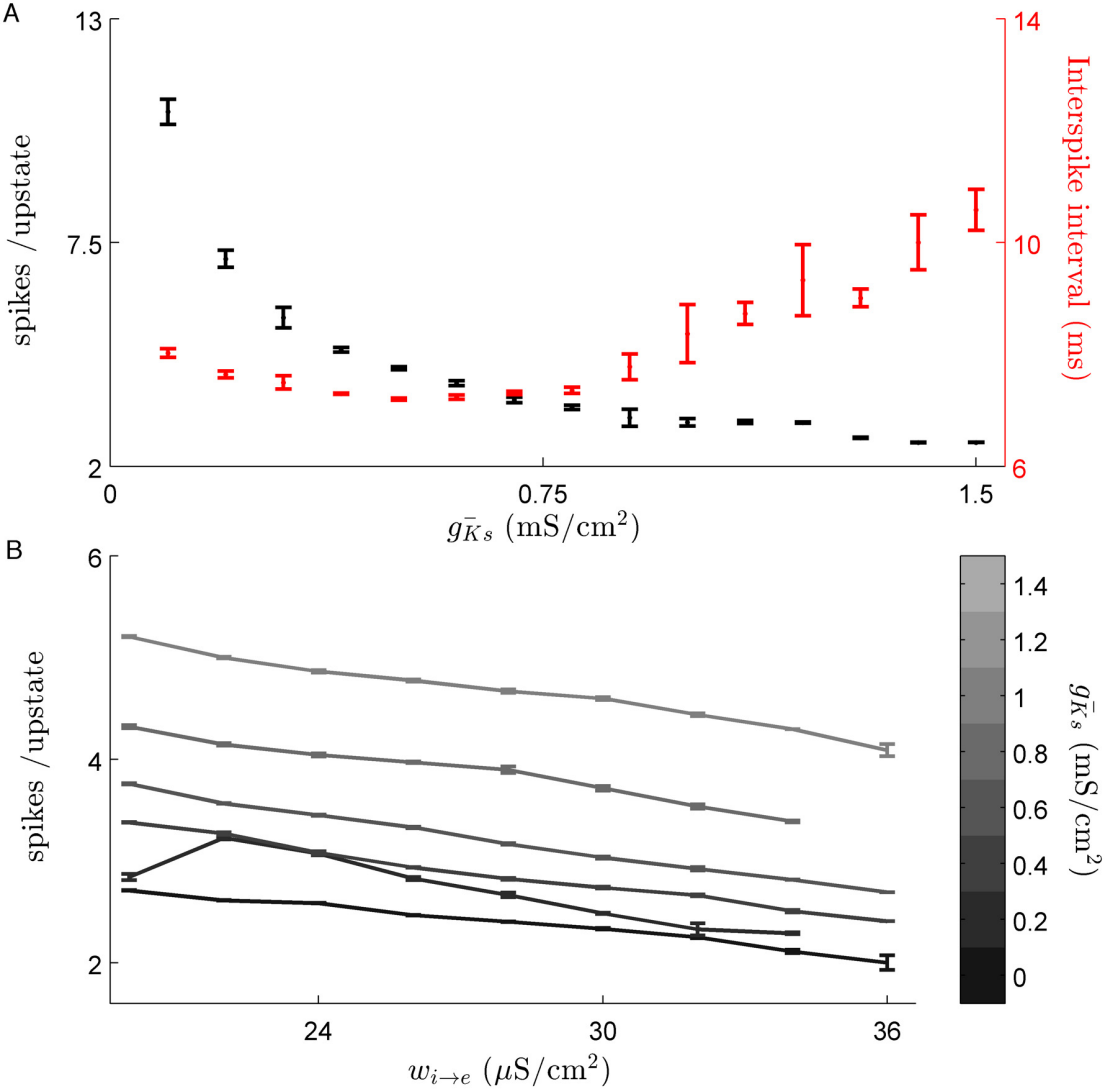
Slow potassium conductance shapes upstate dynamics of individual neurons. (*A*.) For very low levels of $\bar{g_{Ks}}$ individual upstates of neurons last for a longer and more variable number of spikes (*black* *data* *series*). The adaptive effect of the slow potassium conductance is shown by the large variation in ISI for $\bar{g_{Ks}}$ values above 0.75 (*Red* *data* *series*). Increasing $\bar{g_{Ks}}$ reduced the number of spikes per upstate to about 3. Data is shown for *w*_*ie*_ = 24 *μS*/*cm* ^2^ and error bars represent standard deviation. (*B*.) Increasing inhibitory strength, while decreasing wave speed maintains a stable number of spikes per upstate, with average number not changing by more than one (data are mean ± s.e.m).

**Fig 4:**
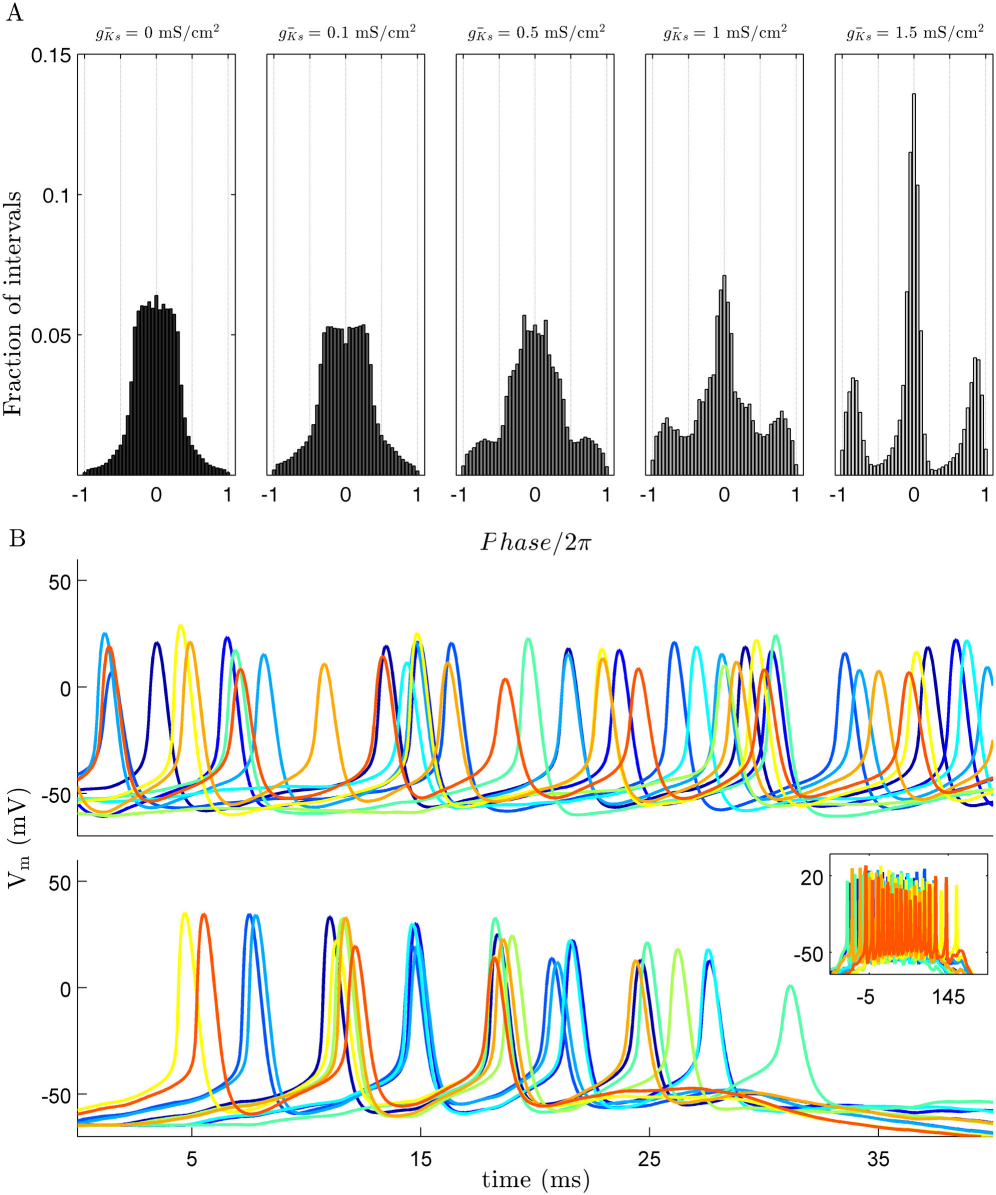
Slow potassium current regulates synchrony through PRC modulation. (*A*.) An increase in spike synchrony within upstates corresponds to the shift from a type 1 to type 2 PRC which occurs at high levels of $\bar{g_{Ks}}$ as indicated by the distribution of inter-spike intervals. Time is shown as normalized phase based on the average period of firing during an upstate and the colors of the bar graphs corresponds to the PRCs shown in Fig 1. (*B*.) Characteristic voltage traces for 10 neighboring cells during an upstate for $\bar{g_{Ks}}=0.1$ mS/cm^2^ *top* and $\bar{g_{Ks}}=1.5$ mS/cm^2^ *bottom*. Each cell is represented by a different color. For both conditions are shown on a 40 ms time scale and the inset shows the entire upstate for the $\bar{g_{Ks}}=0.1$ mS/cm^2^. Data shown here is for *w*_*i* → *e*_ = 24 *μ*S/cm^2^.

From the single cell perspective, the level of SFA has the largest effect on the length of an upstate. Scanning $\bar{g_{Ks}}$ between 0.1 and 1.5 mS/cm^2^ results in a reduction of the number of spikes per upstate. This reduction of spike number corresponds to an increase of both the length and variability of inter-spike intervals (ISIs) within an upstate (Fig 3A). Increasing inhibitory strength has a less dramatic effect on the length of an upstate. For a given value of $\bar{g_{Ks}}$ increasing inhibition reduces the average number of spikes per upstate in a linear fashion, independent of $\bar{g_{Ks}}$ (Fig 3B). While SFA level and E/I balance define the character of neuron upstates, PRC modulation regulates synchrony within the upstate independently (Fig 4). For $\bar{g_{Ks}}$ values large enough to yield traveling waves, but too low to shift the PRC to type 2 ($\bar{g_{Ks}}$ = 0.1 mS/cm^2^ in the figure), spike synchrony between cells with overlapping upstates is low and comparable to levels of synchrony during stationary bump dynamics. In both these cases the ISI interval is approximately uniform ∈ [−*π*, *π*] (Fig 4A; left panels). Increasing $\bar{g_{Ks}}$ to the point where the PRC shifts from type 1 to type 2 leads to high synchrony within the upstates as indicated by an increased observation of ISIs close to 0 or 2*π* and a corresponding decrease at ±*π* (Fig 4A; right panels).

**Fig 5:**
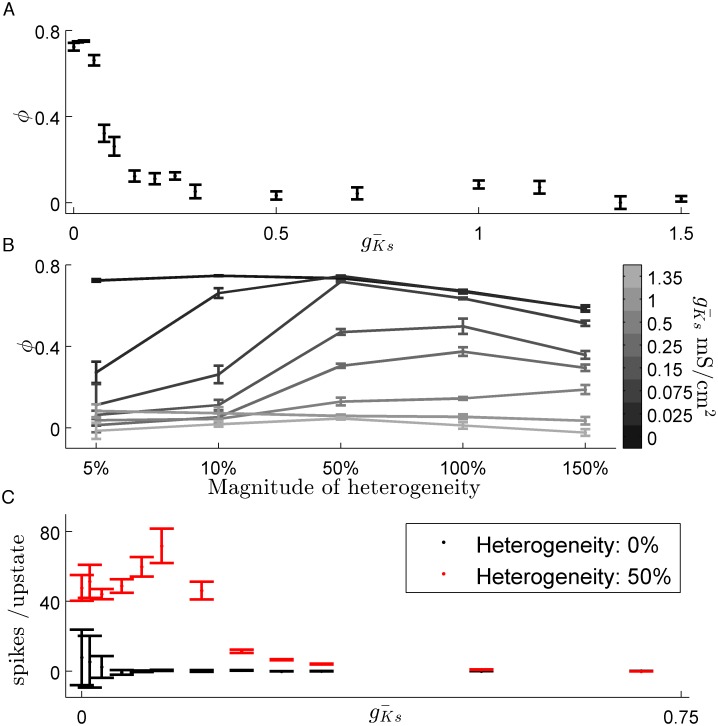
Reducing slow potassium conductance increases network sensitivity to heterogeneities in synaptic coupling. (*A*.) The transition from stationary to moving bump dynamics is demonstrated by *ϕ*, normalized preference for heterogeneity. Increasing $\bar{g_{Ks}}$ rapidly reduces the preference for an area with a 10% increase in strength of recurrent excitatory connections. (*B*.) Significantly enhanced heterogeneities are able to act as an attractor of network activity even for networks with high levels of SFA with a significant preference apparent up to $\bar{g_{Ks}}$ = 0.75 mS/cm^2^. (*C*.) Network preference manifests as longer upstates within the heterogeneous zone than outside it. (data are mean ± s.e.m).

It is known from other studies that in a stationary bump regime, dynamics can be pinned to a specific region by enhanced recurrent excitation [20, 21]. We used this effect to map the transition between local and global dynamics (Fig 5A). We defined *ϕ* (see methods section), as a proxy for the network tendency to localize the dynamics. Increasing $\bar{g_{Ks}}$ from zero rapidly decreases localization with a 50% decrease in *ϕ* occurring within a range of 0.25 mS/cm^2^.

An important function of neural networks is the ability to recognize and respond to structural features such as information encoded in synaptic weights. To explore this idea, we compared how changes in the SFA level affect preferential activation of a region with enhanced recurrent excitation. This effect has been previously shown to localize stationary bump dynamics in spiking networks [20, 21]. Networks with low levels of SFA were highly sensitive to synaptic heterogeneity, with as little as a 5% increase in synaptic strength being sufficient to localized the activation to the heterogeneity (Fig 5B). Sensitivity to heterogeneity decreases as SFA increases as networks allow wave dynamics, but persists for strong heterogeneities well into the wave regime. For low levels of SFA this effect is driven by upstates lasting significantly longer within the heterogeneous area than outside (Fig 5C). This increase in upstate length falls off quickly, even for levels of $\bar{g_{Ks}}$ where *ϕ* displays a preference. This difference stems from *ϕ* being calculated on a longer time scale.

## Discussion

We have shown that changes in SFA level and E/I balance drive the transitions from stationary to traveling (SFA) and local to global (E/I Balance) behavior. These states arise from an interaction between neural excitability and the network-wide strength of lateral inhibition. The magnitude of SFA is a determining factor in whether or not network activity can be pinned by structural heterogeneities such as recurrent excitation. Our results indicate that large scale spatio-temporal dynamics can be induced by ACh mediated SFA and that neural networks composed of highly excitable cells will be more responsive to synaptic heterogeneities. Additionally, ACh induced changes in SFA level and PRC shape occur over differing values of $\bar{g_{Ks}}$.

In the model we used the changes induced by ACh resemble the dynamical cycles seen in the cortex during sleep. Experiments have shown that *in* *vivo* stimulation of cholinergic neurons can induce the transition from SWS to REM like sleep activity [22, 23]. The low ACh state in this model creates traveling waves of high frequency upstates and quiescent down states, reminiscent of what occurs during SWS. Analysis of EEG data in sleeping humans has identified the slow wave in SWS as a traveling wave originating in the frontal cortex and propagating to the posterior [24]. An interesting and relevant feature of the traveling slow wave is that the origins are stable within individuals. Traveling waves in the conductance based model are sensitive to strong heterogeneities for intermediate values of $\bar{g_{Ks}}$. Experiments have shown that inducing local synaptic potential via transcranial magnetic stimulation can define the orgin of traveling slow waves [6, 7]. These results dovetail nicely with our mechanism of recurrent excitation and SFA modulation highlighting regions with strengthened synaptic connectivity.

This model replicates two cellular effects of cholinergic modulation; a reduction of SFA and the shift from a type 1 to a type 2 PRC. The network level consequences of these cellular effect occur over distinct ranges of $\bar{g_{Ks}}$. Previous modeling studies have shown that networks composed of type 1 neural oscillators are generally asynchronous while type 2 networks are highly synchronous [12]. Here we show that neurons with a type 2 PRC are able to synchronize over the short time scale of a single upstate (Fig 4). It is remarkable that type 2 neurons show much higher synchrony than type 1 cells which have much longer to entrain. Type 2 neural oscillators transfer information, measured through spike train correlation, on a much shorter time scale than type 1 oscillators which could explain the difference upstate synchrony [25]. It has been shown previously that network models that learn via spike timing dependent plasticity (SDTP) will strengthen synapses when composed of type 1 neurons, while weakening occurs when component neurons are of type 2 [26].

SWS is critical for memory consolidation, particularly during early stages [27–29]. The changes in both SFA level and in the PRC shape are both likely to play a role in the changes in synaptic strength during SWS, but whether they interact synergistically is unclear and will be the topic of further study. Another important implication of these results is to show how stationary versus traveling dynamics fit into the frameworks proposed by the synaptic homeostasis hypothesis (SHY) [30], which proposes synaptic renormalization during sleep, and the synaptic embossing hypothesis (SEH) [31], in which select circuits are strengthened by synchronous firing during REM in addition to renormalization during SWS. It may be that localized asynchronous activity during REM sleep can further strengthen regions specified by enhanced synaptic strength during waking, while traveling, but synchronous, activity within a globally traveling wave can cause global depotentiation of synapses. This would lead to a large increase in synaptic signal to noise ratio as proposed by SHY [30] while employing a REM dependent dynamical mechanism proposed by SEH [31]. Recent *in* *vitro* and *in* *silico* studies have demonstrated the importance of REM sleep on experience dependent plasticity [32, 33]. The differing $\bar{g_{Ks}}$ ranges for SFA induced local to global and the PRC induced asynchronous to synchronous transitions may account for the importance of SWS to REM transitions in synaptic restructuring recently reported [33]. The interaction between ACh level and inhibitory strength in our model could be functionally significant. The administration of GABAergic drugs (which correspond to higher *w*_*i* → *e*_ values in out model) increases the time spent in SWS and the power in the delta (∼ 1 Hz) range, but does not measurably increase memory consolidation [34]. This may be due to the interaction of the two aforementioned mechanisms, but also to the increased GABA levels changing features of the traveling waves during SWS. It would be interesting to see whether GABA agonists decrease the propagation speed of SWS waves in LFP measurements.

To demonstrate the extent that the spatial properties of upstates are set by E/I balance we sampled parameters that fall outside of normal physiological conditions and only values that fall within the reduced range that yield single bumps produce dynamics representative of sleeping or waking states. During SWS, increased activity of GABAergic projections from the basal forebrain increase both phasic and tonic inhibition within the cortex [35]. Pharmacologically enhancing phasic inhibition, which would skew E/I balance toward inhibition in our model, decreases power in the delta band [36]. Increasing inhibition caused a decrease in the average number of spikes per upstate and narrowed the spatial extent of an upstate, both of which would lead to a decrease in LFP power. On the other hand, increasing tonic inhibition leads to an increase of delta power [36]. This model does not include a representation of tonic inhibition and adding this feature would be a valuable extension of these results. During high ACh conditions a more complicated inhibition conditions exist. While state dependent GABA input from the basal forebrain is reduced, muscarinic agonists increase the amplitude and frequencies of spontaneous inhibitory postsynaptic currents [37]. This enhanced inhibition on its own would increase localization and sensitivity of stationary dynamics. Cholinergic drugs decrease the magnitude of evoked inhibitory input, however [38]. Whether or not these effects lead to a net E/I balance shift is not clear.

**Fig 6:**
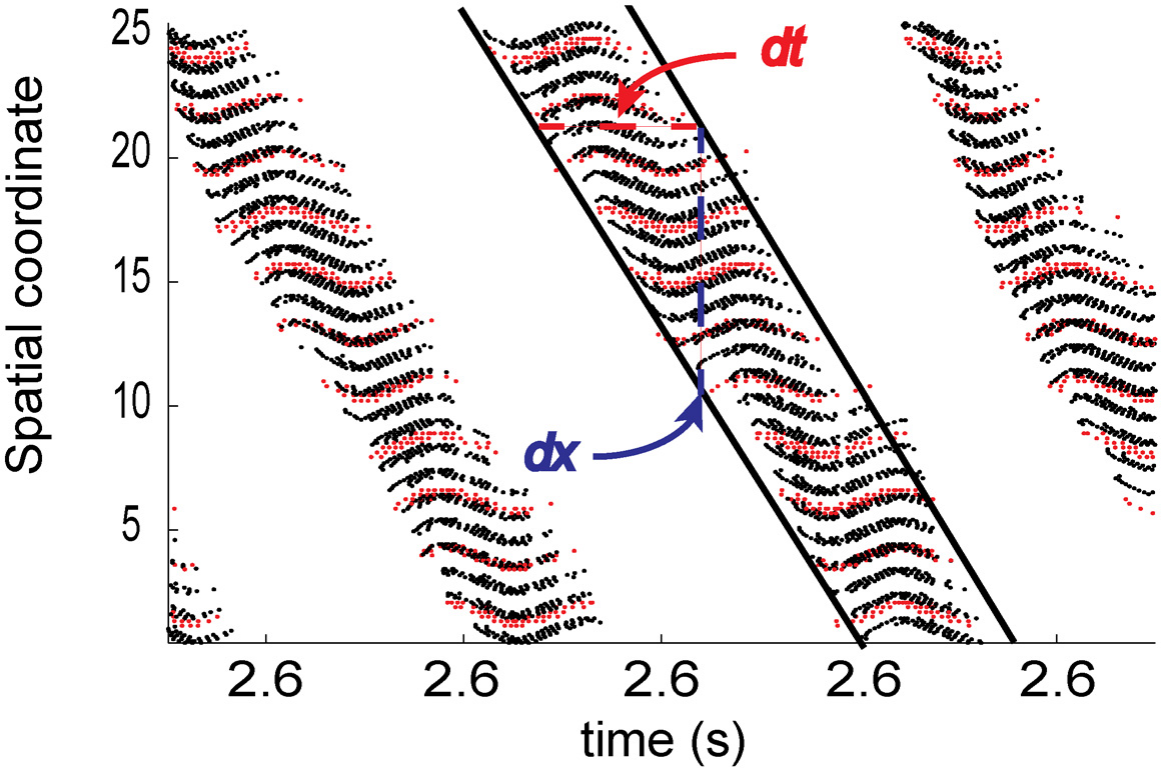
Slow potassium conductance and E/I balance work in concert to shape upstate traveling bump dynamics. The strength of inhibition determines the spatial scope of an active zone, or the space a traveling bump will traverse in a given time (the *dx* shown in blue above). The length of an upstate at any given point in space is governed by the strength of the slow potassium conductance $\bar{g_{Ks}}$ (illustrated by the red *dt* above). These two features form the rough approximation of wavespeed *dx*/*dt*. As in Fig 2., black markers represent spikes from excitatory cells, red markers represent those from inhibitory cells and cells are sorted along the y-axis by spatial coordinate.

When the network is in the stationary state (when $\bar{g_{Ks}}$ is low and SFA is minimal; the high ACh state) the excited region generates large levels of distal inhibition that reduces the likelihood that neurons outside this region will fire. Reducing the strength of inhibition causes a corresponding increase in the likelihood that far away cells will fire, eventually leading to a global high frequency state (Fig 1C). As SFA is increased (when $\bar{g_{Ks}}$ is increased or ACh levels fall) the length of an individual neuron’s upstate becomes limited. As excited cells enter a period of quiescence, neighboring neurons are able to enter an upstate due to a relaxation of distal inhibition. This relaxation increases the spatial extent of cells that are in an upstate at the same time. These two factors affect the character of spatio-temporal dynamics by effectively setting the two components of wave speed, *dx*/*dt* (Fig 6). The strength of inhibition sets *dx*, with lower levels increasing its magnitude (and thus total wave speed as well). A large amount of SFA shortens *dt* which drives large increases in wave speed. This notion also explains how synaptic heterogeneity (i.e. enhanced recurrent excitation) acts to pin activity. When the excited region passes over areas with increased excitatory coupling the recurrent excitation is able to reduce the effects of SFA on neurons causing an increase in *dt* when activity is within this area decreasing the propagation of excitation.

Stationary bump dynamics have long been used as a model of working memory [39–41]. In this model, the location of excitation preserves the location of a transient input and synaptic heterogeneities stabilize bump location [20, 21]. Recent experimental results have demonstrated both the importance of stationary bumps in attention tasks [5] and the importance of the muscarinic system in this state [3]. Our results suggest that cholinergic modulation of SFA primes a network to focus on incoming information, providing a mechanism for ACh’s role in attention [42].

In neural field models, the conditions that lead to the formation of stationary bumps and traveling waves have been well documented [43, 44]. Lateral inhibition is necessary for the formation of stationary bumps and traveling waves [17, 45], and is critical for our results. While our results hold when the range of inhibition is reduced from global, we do need the radius of inhibitory connections to be larger than that of excitation. In fact, we do not believe that traveling waves can form unless the inhibitory range is larger than that of excitatory connections. While our scheme is supported by some experimental evidence [14, 15], other results have failed to find lateral inhibition as a model for cortical connectivity [46, 47]. While it is possible that the range of synaptic coupling for inhibitory interneurons is shorter than that of excitatory cells, electrical synapses (i.e. gap junctions) could broaden the scope of inhibition. Furthermore, dynamic regulation of gap junctions could allow for network topology to vary according to the requirements of a particular activity regime [48].

In addition to lateral inhibition, SFA also induces traveling waves in both neural field models and in other more complex spiking networks [49, 50]. In other models, SFA causes linearization of the f-I curve in a similar manner as $\bar{g_{Ks}}$[51, 52] and the mechanism we describe here is likely a general phenomenon in the formation of waves in adapting networks. Analytical results from neural fields have related higher thresholds (the level of input required to generate action potentials) to decreased propagation speed of traveling waves [44, 49]. This may disagree with our results, which are that threshold and wave speed increase with $\bar{g_{Ks}}$. The threshold in neural field models may relate more to E/I balance in our system than to the threshold for spiking of individual neurons. It is important to note that the model we use does not address other important facets of muscarinic neuromodulation such as resting potential and leak conductances [53], synaptic strength [54], and both Ca^2+^ and Na^+^ dependent K^+^ currents [4, 50], all of which likely play a role in the formation of spatiotemporal dynamics.

That SFA and PRC modulation take place over different ranges of $\bar{g_{Ks}}$ allows for three general regimes within networks of this type: localized asynchronous, traveling asynchronous, and traveling synchronous. It is clear from our results that these regimes differ in sensitivity to synaptic heterogeneity (decreasing from localized asynchronous to traveling synchronous) but whether they represent distinct functional states, especially regarding processes such as memory and synaptic homeostasis, need further experimental and computation work.

## Supporting Information

S1 VideoNetwork dynamics for inhibitory fraction of 13% and $\bar{g_{Ks}}=$ 0mS/cm^2^.(AVI)

S2 VideoNetwork dynamics for inhibitory fraction of 13% and $\bar{g_{Ks}}=$ 1.5mS/cm^2^.(AVI)

S3 VideoNetwork dynamics for inhibitory fraction of 22% and $\bar{g_{Ks}}=$ 0mS/cm^2^.(AVI)

S4 VideoNetwork dynamics for inhibitory fraction of 22% and $\bar{g_{Ks}}=$ 1.5mS/cm^2^.(AVI)

S5 VideoNetwork dynamics for no periodic boundaries and $\bar{g_{Ks}}=$ 1.5mS/cm^2^.(AVI)

S1 Fig Distribution of bursting values. High values of the bursting measure, B, indicate highly synchronous firing. The distribution of B is bimodal and a value of 0.7 (red line) was chosen to exclude highly synchronous dynamics because it divides the distribution.
(TIF)

S2 Fig Examples of observed dynamics. A broad array of dynamics were observed. The phase cartoon from Fig 1D is included with raster plots displaying dynamics. Numbers indicate the following: (1) quiescent (2) mixed dynamics, (3) stationary bump (4) traveling bump (5) global high frequency activity (6) multiple interacting bumps (7) planar wave (8) global burst. Note two examples of mixed dynamics (2) were included to show that traveling waves, stationary bumps, highly synchronized bursts, and quiescence arise during the course of a simulation.
(TIF)

S1 Table Table of parameters. Values of the neural parameters were adopted from [12].
(PDF)

S1 File Simulation code. (CPP)
