# Supplementary figures and images for: Formation and Dynamics of Waves in a Cortical Model of Cholinergic Modulation
Source: PLoS Comput Biol. 2015 Aug 21;11(8):e1004449. doi: 10.1371/journal.pcbi.1004449 (PMC4546669; doi:10.1371/journal.pcbi.1004449)

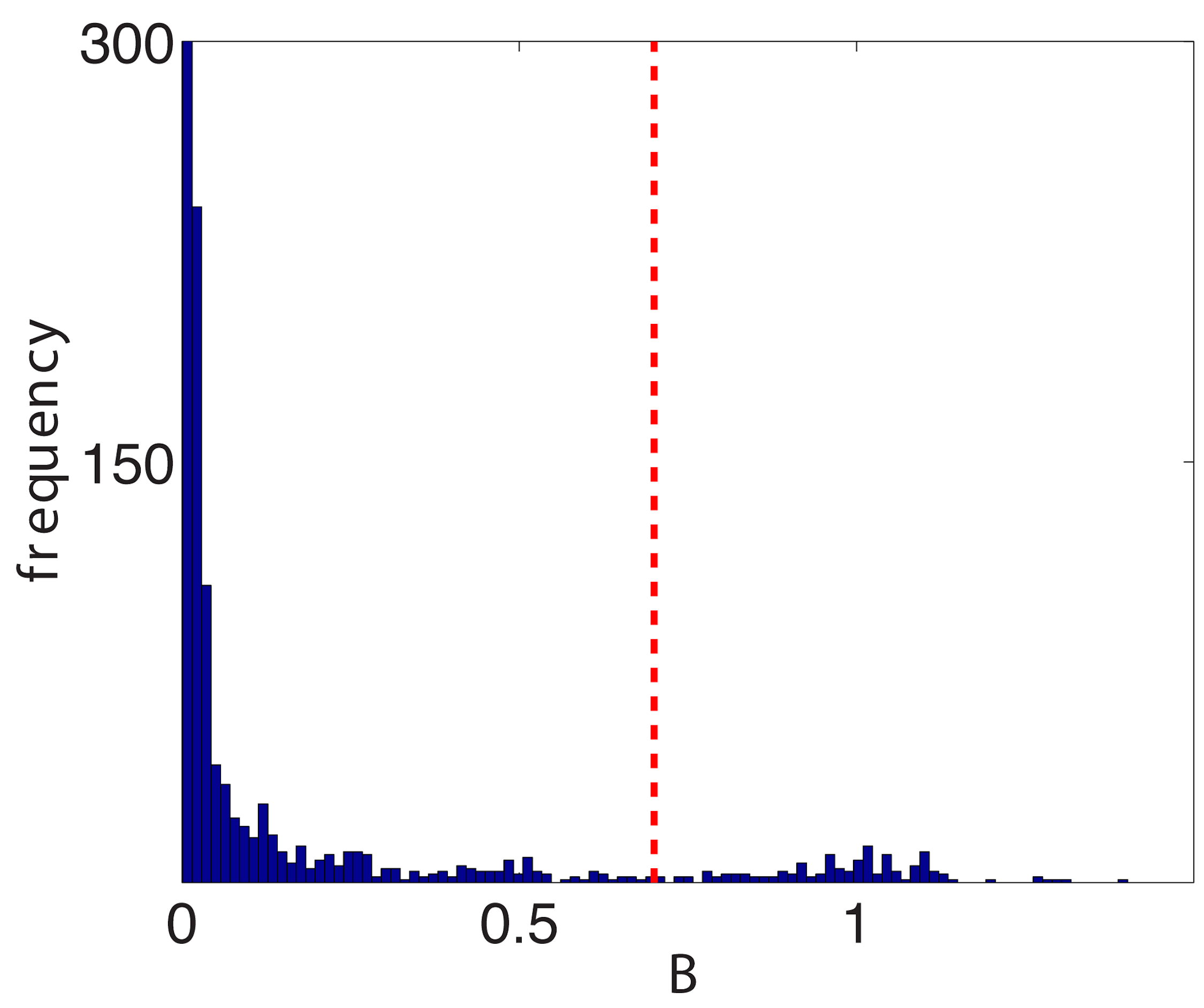

Supplement: S1 Fig — High values of the bursting measure, B, indicate highly synchronous firing. The distribution of B is bimodal and a value of 0.7 (red line) was chosen to exclude highly synchronous dynamics because it divides the distribution. (TIF) [file pcbi.1004449.s006.tif]

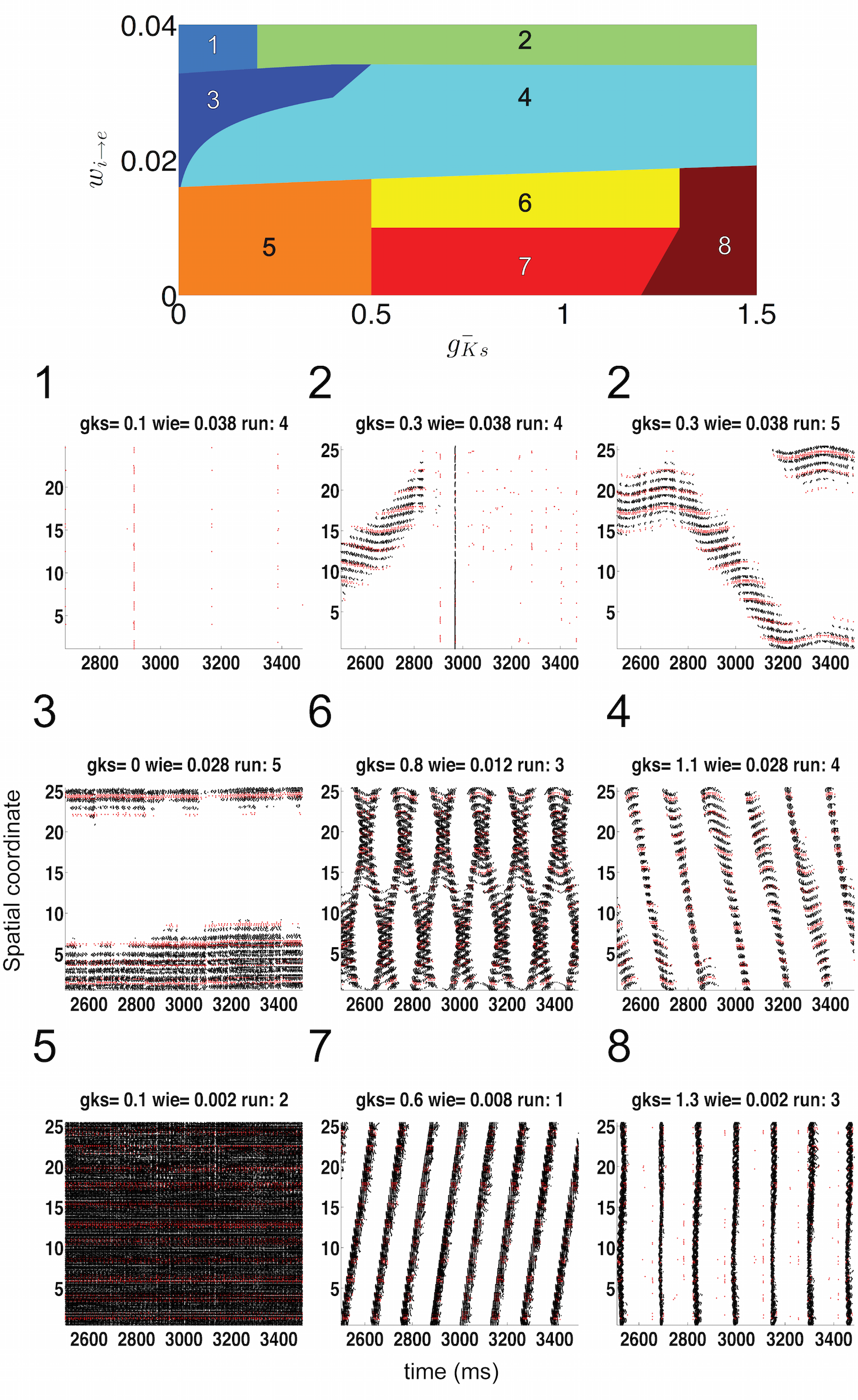

Supplement: S2 Fig — A broad array of dynamics were observed. The phase cartoon from Fig 1D is included with raster plots displaying dynamics. Numbers indicate the following: (1) quiescent (2) mixed dynamics, (3) stationary bump (4) traveling bump (5) global high frequency activity (6) multiple interacting bumps (7) planar wave (8) global burst. Note two examples of mixed dynamics (2) were included to show that traveling waves, stationary bumps, highly synchronized bursts, and quiescence arise during the course of a simulation. (TIF) [file pcbi.1004449.s007.tif]
