# Supplementary material for: Formation and Dynamics of Waves in a Cortical Model of Cholinergic Modulation
Source: PLoS Comput Biol. 2015 Aug 21;11(8):e1004449. doi: 10.1371/journal.pcbi.1004449 (PMC4546669; doi:10.1371/journal.pcbi.1004449)
Supplement: S1 Table — Values of the neural parameters were adopted from [12]. (PDF) [file pcbi.1004449.s008.pdf]

**Neural parameters**			
c_m	1.0 $\mu F/cm^2$	$g_{\bar{K}s}$	0-1.5 mS/cm^2
$g_{\bar{N}a}$	24.0 mS/cm^2	g_L	0.02 mS/cm^2
$g_{\bar{K}dir}$	3.0 mS/cm^2	E_{Na}	55.0 mV
E_K	-90.0 mV	E_L	-60.0 mV
α_m	-30 mV	α_h	53 mV
α_n	- 30 mV	α_s	-39 mV
β_m	9.5 mV	β_h	7 mV
β_n	10 mV	β_s	5 mV
γ_h	40.5 mV	γ_n	27 mV
ε_h	6 mV	ε_n	15 mV
D_h	2.78	D_n	1.85
$V_{threshold}$	-20 mV		
**Synapse parameters**			
τ_F	0.3 ms	τ_S	3 ms
E_e	0 mV	E_i	-75 mV
τ_D	-75 mV		
**Network parameters**			
$w_{e\to e}$	20 $\mu S/cm^2$	$w_{i\to e}$	0-38 $\mu S/cm^2$
$w_{e\to i}$	20 $\mu S/cm^2$	$w_{i\to i}$	20 $\mu S/cm^2$
L	25 $units$	$Grain_e$	1 $unit$
$Grain_i$	2.27 $units$	k_{ee}	16
k_{ei}	4		
